# Effect of Shisha (Waterpipe) Smoking on Lung Functions and Fractional Exhaled Nitric Oxide (FeNO) among Saudi Young Adult Shisha Smokers

**DOI:** 10.3390/ijerph110909638

**Published:** 2014-09-17

**Authors:** Sultan Ayoub Meo, Khaled Ahmed AlShehri, Bader Bandar AlHarbi, Omar Rayyan Barayyan, Abdulrahman Salem Bawazir, Omar Abdulmohsin Alanazi, Ahmed Raad Al-Zuhair

**Affiliations:** Department of Physiology, College of Medicine, King Saud University, P.O. Box 2925, Riyadh 11461 Saudi Arabia; E-Mails: K_a_Z_h@hotmail.com (K.A.A.); Bdr.alharbi.431@gmail.com (B.B.A.); Mr.omar.b@hotmail.com (O.R.B.); Dr.a.bwz@gmail.com (A.S.B.); Dr-3umar92@hotmail.com (O.A.A.); Ahmed-alzuhair@hotmail.com (A.R.A.-Z.)

**Keywords:** lung function, fractional exhaled nitric oxide, Shisha smoking

## Abstract

Shisha (waterpipe) smoking is becoming a more prevalent form of tobacco consumption, and is growing worldwide, particularly among the young generation in the Middle East. This cross-sectional study aimed to determine the effects of shisha smoking on lung functions and Fractional Exhaled Nitric Oxide (FeNO) among Saudi young adults. We recruited 146 apparently healthy male subjects (73 control and 73 shisha smokers). The exposed group consisted of male shisha smokers, with mean age 21.54 ± 0.41 (mean ± SEM) range 17–33 years. The control group consisted of similar number (73) of non-smokers with mean age 21.36 ± 0.19 (mean ± SEM) range 18–28 years. Between the groups we considered the factors like age, height, weight, gender, ethnicity and socioeconomic status to estimate the impact of shisha smoking on lung function and fractional exhaled nitric oxide. Lung function test was performed by using an Spirovit-SP-1 Electronic Spirometer. Fractional Exhaled Nitric Oxide (FeNO) was measured by using Niox Mino. A significant decrease in lung function parameters FEV1, FEV1/FVC Ratio, FEF-25%, FEF-50%, FEF-75% and FEF-75-85% was found among shisha smokers relative to their control group. There was also a significant reduction in the Fractional Exhaled Nitric Oxide among Shisha smokers compared to control group.

## 1. Introduction

Shisha smoking is known by a various names, including waterpipe, narghile, arghile, ghoza, borry, shui yan dai, hubble bubble and hookah smoking [1]. Shisha smoking originated in India, South Africa, Persia, and Ethiopia and currently is a common practice in Arab countries [2]. Its use is frequent among the youth, high income and urban population, especially in posh localities [3]. It has steadily been spreading among the various age groups, but especially among adolescents around the world, and gained more popularity in the Middle East in the form of Shisha cafe culture. The prevalence of Shisha smoking ranges from 5%–17% in American youths, 6%–34% among Middle Eastern adolescents [4], and in some studies the prevalence of Shisha smokers has been reported to be up to 44.3% among immigrants in the U.S. [5].

Shisha smoke contains large quantities of flavored nicotine, fine and ultrafine PM, carbon monoxide, polycyclic aromatic hydrocarbons, volatile aldehydes, phenolic compounds and carcinogenic PAH [6,7,8,9,10,11], and heavy metals, including arsenic and lead. Shisha smokers use tobacco flavored with apple, plum, coconut, mango, mint, strawberry and cola, which makes the act of smoking more attractive, sweet and aromatic than cigarette smoking.

The most common types of tobacco used in the Shisha smoking are: Maassel, Ajami, Tumbak and Jurak [12]. The nicotine content of water pipe tobacco is 2%–4% and carbon monoxide concentration is 0.34%–1.40%. Shisha smoking sessions usually last for 20–80 min [13]. The smokers are thus exposed to much more smoke over a longer period of time. The smoker takes 50–200 puffs; inhales 0.15–1 L of smoke in one session of Shisha which is equivalent to smoke of about 100 cigarettes [12,13].

Lung function tests are a widely used tool to describe the effects of obstruction or restriction on lung function. It is a powerful diagnostic tool that plays a significant role in the diagnosis of early lung damage. It is also used to monitor the therapeutic efficacy of various treatment regimens and the course of the disease. The spirometric parameters have gained more popularity when it has been reported that Forced Vital Capacity (FVC), Forced Expiratory Volume in 1 s (FEV1), and FEV1/FVC Ratio are essential for the diagnosis of obstructive and restrictive respiratory illness [14]. Similarly, Measurement of Fractional Nitric Oxide (NO) in exhaled breath (FeNO) is a simple, safe, reliable and non-invasive tool in assessing the severity of pulmonary inflammation and asthma [15].

Cigarette smoking-related research and control efforts have generally been focused on traditional cigarettes, while little research exists on Shisha smoking and most of the literature fails to consider the physiological parameters including age, height, weight, gender, ethnicity and socio-economical matching between the groups. In order to address the emerging health risk, the present study aimed to determine the effects of Shisha smoking on lung function and Fractional Exhaled Nitric Oxide (FeNO) among young Saudi adults.

## 2. Subjects and Methods

### 2.1. Subjects

All subjects gave their informed consent for inclusion before they participated in the study. The study was conducted in accordance with the Declaration of Helsinki, and the protocol was approved by the Ethics Committee of College of Medicine Research Centre, King Saud University, Riyadh, KSA (CMED-305-MB7).

This cross sectional study was conducted in the Department of Physiology, College of Medicine, King Saud University, Riyadh, Saudi Arabia during the period January 2013‒Decemeber 2013. Between the groups age, height, weight, gender, ethnicity and socioeconomic status factors were considered to estimate the impact of Shisha smoking on lung function and fractional exhaled nitric oxide. For this study, 146 participants (73 Shisha smokers and 73 non Shisha smokers) were selected. A detailed interview of the subjects was conducted, followed by history taking and clinical examination to determine whether to include him in the study or not. All the participants were questioned with regards to cigarette, Shisha smoking and other tobacco product consumption. The Shisha exposed group consisted of 73 volunteer, male subjects. It consisted of university students, clerks, secretarial staff and salespersons. The control group was selected in a similar way. Initially, 100 subjects were interviewed, clinical history and examination were conducted, and finally 73 volunteer, healthy men were selected. The control group primarily consisted of university students plus receptionists, secretaries, porters and research assistants.

### 2.2. Exclusion Criteria

Subjects with known cases of gross anemia, diabetes mellitus, chronic obstructive pulmonary diseases, bronchial asthma, malignancy, drug addicts and tobacco smokers (other than Shisha) were excluded. The subjects who were regularly exercising vigorously or working in any industry which generates dust or fumes were also excluded from the study [16].

### 2.3. Methods: Lung Function Test (Spirometry)

To assess Lung Function in subjects spirometry was performed using a SPIROVIT SP-1 (Schiller AG, Baar, Switzerland) instrument. The following lung function parameters were included: Forced Vital Capacity (FVC), Forced Expiratory Volume in first second (FEV1), Forced Expiratory Ratio (FEV1/FVC%), Peak Expiratory Flow (PEF), Forced Expiratory Flow (FEF-25%), Forced Expiratory Flow (FEF-50%), Forced Expiratory Flow (FEF-75%), Forced Expiratory Flow (FEF-25-75%) and Forced Expiratory Flow (FEF-75-85%). The defined techniques in executing various lung function tests for the present study were based on the operation manual of instrument with special reference to the official statement of the American Thoracic Society of Standardization 2005 [17]. After taking a detailed history and anthropometric data, the subjects were informed about the whole maneuver. The subjects were encouraged to practice this maneuver before doing the pulmonary function test. The participants were instructed three times, and the test was performed with the subject in the standing position with a nose clip and best values were taken. The subject was asked to inhale to the maximum and exhale completely as fast as possible and make sure to get all the air out of the lungs. A new disposable sterile mouth piece was used for each participant to prevent any cross infection.

### 2.4. Fractional Exhaled Nitric Oxide (FeNO)

Fractional Exhaled Nitric Oxide (FeNO) concentration was measured by using a Niox Mino unit, (Aerocrine, Solna, Sweden). The FeNO device was pre-calibrated for a predetermined life span (300 measurements) by the manufacturer; hence the device did not require re-calibrations in the field. Tests were carried out at a fixed time of the day to minimize the diurnal variation of FeNO concentration [18]. The defined techniques in executing FeNO test for the present study were based on the operation manual of the instrument with special reference to the official statement of the American Thoracic Society/ERS Standardization procedure for FENO measurements [19]. After taking a detailed history and anthropometric data, the subjects were informed about the whole maneuver. All measurements of the Shisha smokers were conducted in the adjacent room of Shisha cafe and for control group all measurements were performed in the Physiology lab of College of Medicine, KSU. The tests were performed with the subject in the standing position while using a nose clip. The results were recorded through a laptop computer attached to the Niox Mino, Aerocrine, Solna, Sweden.

### 2.5. Statistical Analysis

The data were entered into the computer and analyzed by using the Statistical Package for Social Sciences (SPSS for Windows, version 20.0). Unpaired Student’s *t*-test (parametric test) was applied to test the difference of the means between the two quantitative variables. The level of significance was assumed at *p* < 0.05.

## 3. Results and Discussion

Table 1 summarizes the comparison of the anthropometric variables and lung function parameters between Shisha smokers and their control group. The mean age of Shisha smokers was 21.54 ± 0.41 (mean ± SEM) years, height 172.68 ± 0.76 (mean ± SEM) cm, weight 76.26 ± 2.39 (mean ± SEM) kg. However, for control group the mean age 21.36 ± 0.19 (mean ± SEM) years, height 173.71 ± 1.03 (mean ± SEM) cm and weight was 72.84 ± 1.48 (mean ± SEM) kg. There was a significant decline in the lung function parameters of Shisha smokers including FEV1 (*p* = 0.0001), FEV1/FVC Ratio (*p* = 0.0001), FEF-25% (*p* = 0.005), FEF-50% (*p* = 0.0001), FEF-75% (*p* = 0.0001), and FEF-75-85% (*p* = 0.010) compared to their age, height, weight and ethnicity matched non-Shisha smoker subjects.

However, there was no reduction in FVC, FEF-25-75% and PEF. Moreover, in the present study, we found that fractional exhaled nitric oxide (FeNO) was also significantly decreased in Shisha smokers (*p* = 0.022), compared to their control subjects. The mean duration of Shisha smokers was 2.97 ± 0.22 years (mean ± SEM), range 1–8 years.

Table 2 presents the mean values of anthropometric variables and FeNO level for the Shisha smokers and the control group. The statistical comparisons of the matching variables including age, height and weight, ethnicity and socioeconomic status were similar for both groups. Mean FeNO of Shisha smokers was 23.97 ± 2.12 ppb compared to their control group 31.38 ± 2.38 ppb. The FENO was significantly decreased in Shisha smokers (*p* = 0.022) compared to their control group.

**Table 1:** Comparison of various lung function parameters between Shisha Smokers compared to their matched control group.

Parameters	Shisha Group (*n* = 73)	Control Group (*n* = 73)	*p* Values
Age (years)	21.54 ± 0.41	21.36 ± 0.19 years	0.697
Height (cm)	172.68 ± 0.76	173.71 ± 1.03 cm	0.427
Weight (kg)	76.26 ± 2.39	72.84 ± 1.48 kg	0.227
FVC (L)	5.76 ± 0.21	5.54 ± 0.11	0.351
FEV1 (L)	3.80 ± 0.12	4.49 ± 0.073	0.0001
FEV1//FVC (%)	69.34 ± 1.87	82.83 ± 1.29	0.0001
FEF-25% (L/sec)	6.04 ± 0.25	6.93 ± 0.18	0.005
FEF-50% (L/sec)	3.13 ± 0.19	5.25 ± 0.17	0.0001
FEF-75% (L/sec)	1.13 ± 0.13	2.56 ± 0.13	0.0001
FEF-25-75% (L/sec)	6.86 ± 2.23	4.53 ± 0.19	0.293
FEF-75-85% (L/sec)	1.21 ± 0.24	1.91 ± 0.11	0.010
PEF (L/sec)	7.29 ± 0.22	7.32 ± 0.18	0.895

Notes: Values are presented in Mean ± SEM

**Table 2:** Comparison of Fractional Exhaled Nitric Oxide (FeNO) between Shisha smokers compared to their matched control group.

Parameters	Shisha Group	Control Group	*p* Values
Age (years)	21.54 ± 0.41	21.36 ± 0.19	0.697
Height (cm)	172.68 ± 0.76	173.71 ± 1.03	0.427
Weight (kg)	76.26 ± 2.39	72.84 ± 1.48	0.227
FeNO (ppb)	23.97 ± 2.12	31.38 ± 2.38	0.022

Note: Values are presented in Mean ± SEM.

## 4. Discussion

### 4.1. Main Findings

Tobacco smoking is a global public health risk causing more than 6 million deaths each year; approximately one person dies every six seconds due to tobacco [20]. Tobacco is commonly smoked in different ways including cigarette, cigar, and Shisha. Presently, Shisha smoking is gaining popularity among the young, high income and urban population of the world. It is becoming a new tobacco pandemic and severely affecting the indoor air quality and involves severe health risks [11]. Shisha smoking produces large amount of toxic ultrafine particles and carry similar health risks as smoking cigarettes [7]. In the present study, we determined the effects of Shisha smoking on lung functions and Fractional Exhaled Nitric Oxide (FeNO) among Saudi young adults. We found a significant reduction in lung function parameters FEV1, FEV1/FVC%, FEF-25%, FEF-50%, FEF-75%, FEF-75-85% in Shisha smokers relative to their matched control group. There was also a significant reduction in the Fractional Exhaled Nitric Oxide (FeNO) in Shisha smokers compared to control group.

### 4.2. Lung Function Tests

Lung function test is ideally suited to describe the obstructive and restrictive pattern of respiratory diseases in various occupational and environmental settings [21,22,23]. It is an essential part of a respiratory function evaluation and is used to categorize the nature, severity and progression of respiratory diseases. In the present study, we found a significant reduction in lung function parameters FEV1, FEV1/FVC Ratio, FEF-25%, FEF-50%, FEF-75% and FEF-75-85% in Shisha smokers relative to their matched control group.

Al-Fayez *et al.* [24] reported the effects of Shisha smoking on pulmonary function test values. They found that FEV1 and FVC mean values of male Shisha smokers were significantly lower than those of non-smokers. Pulmonary function measurements demonstrate a decline pattern with age of Shisha smokers particularly in the age group 20–49. They conclude that Shisha smoking produce adverse effects on the pulmonary functions and increase the risk of developing obstructive airway disease. Similarly, in the present study we found a significant decline in lung function parameters and the pattern of lung function impairment was obstructive airway disease. This obstructive defect is documented in relatively young individuals, and after a relatively short duration of waterpipe smoking. This finding contradicts the general public belief that waterpipe smoking is safer than cigarette smoking.

Hakim *et al.* [25] reported the acute effects of Shisha (waterpipe) smoking on pulmonary function test results. There was a decrease in forced expiratory flow between FEF-25% and FEF-75% of FVC, peak expiratory flow rate. They show that waterpipe smoking causes biologic changes that might result in marked health problems. Likewise, Hawari *et al.* [26] conducted a study on waterpipe tobacco smokers with mean age 20.4 years, they found that forced expiratory flow over the middle half of the forced vital capacity (FEF50%) was decreased among waterpipe tobacco smokers. Similarly, in the present study we found a significant reduction in lung function parameters FEV1, FEF-25%, FEF-50%, FEF-75% and FEF-75-85% in Shisha smokers relative to their matched control group.

Raad *et al.* 2011 [27] conducted a meta-analysis comparing water-pipe smokers with nonsmokers for spirometric measurements of FEV1, FVC, and FEV1/FVC Ratio. Waterpipe tobacco smokers were associated with significant reduction in FEV1, FVC and lower FEV1/FVC Ratio. Correspondingly, in the present study, in addition to other parameters, we found a significant reduction in FEV1 and FEV1/FVC Ratio in Shisha smokers relative to their matched control group.

Schünemann *et al.* [28] reported that waterpipe smokers were associated with a significant reduction of FEV_1_ and a trend toward lower FVC and FEV_1_/FVC Ratio. Boskabady *et al.* [29] showed a profound effect of waterpipe smoking on lung function values. They found that FVC, FEV1, MMEF, PEF, MEF-75, MEF-50, MEF-25 in waterpipe smokers were significantly lower than the corresponding values in non-smokers. Concurrently, in the present study, we found a significant reduction in FEV1, FEV1/FVC Ratio, FEF-25%, FEF-50%, FEF-75% and FEF-75-85% in Shisha smokers relative to their matched control group.

### 4.3. Fractional Exhaled Nitric Oxide (FeNO)

Fractional exhaled nitric oxide (FeNO) is a non-invasive, widely used method for assessing the inflammatory status of the lungs with airway disease [30,31,32,33]. It has achieved great consideration in the diagnosis of respiratory diseases and become highly popular parameter both in respiratory researchers and clinicians. It has recently been standardized by both American Thoracic Society and European Respiratory Society (ATS/ERS-2005) [33]. FeNO is an essential marker of eosinophilic airway inflammation, markedly elevated in bronchial asthma [32], chronic airway inflammation and allergic rhinitis [33]. It is recognized as a precise, reproducible and noninvasive diagnostic test for airway disease [34]. It is influenced by variety of patho-physiological processes in the upper and lower airways, tobacco smoking [32] and working exposure in environmental and occupational industries which cause air pollution [16,17,35,36,37,38,39].

She *et al.* [40] found the significantly increased risk of COPD in Chinese waterpipe smokers compared to never-smoking controls. Similarly based on the results of present study we found that Shisha smoking is associated with chronic obstructive pulmonary diseases (COPD).

The main finding of the present study is that Shisha smoking is associated with a reduction in FeNo levels. Few studies have reported reduced levels of FeNO after both short- and long-term cigarette smoking [41]. Possible mechanisms include down regulation of endothelial and inducible nitric oxide synthase, and rapid conversion of nitric oxide to peroxynitrite by reactive oxygen and nitrogen species [42,43].

Smoking is associated with a reduction in exhaled nitric oxide (FeNO) levels. There is, however, limited knowledge relating to the smoking-induced changes in production of NO in different compartments of the airways [44]. In the present study, we found a significant reduction in Fractional Exhaled Nitric Oxide (FeNO) in Shisha smokers compared to control group. In parallel to our findings, Hakim *et al.*, reported the effects of waterpipe smoking on fractional exhaled nitric oxide (FeNO) levels. There was a decrease in FeNO levels. They showed that waterpipe smoking causes biologic changes that might result in marked health problems.

### 4.4. What This Study Adds

Shisha (waterpipe) smoking is a swiftly growing global epidemic and causes serious complications. Shisha smoking significantly decreases the lung function parameters and Fractional Exhaled Nitric Oxide (FeNO) compared to their matched control. It affects the young subjects even after a relatively short duration of exposure. The present study is one of the first studies to investigate effect of Shisha smoking on lung functions and exhaled nitric oxide. This research provides awareness to the community and to the health officials regarding the effects of smoking Shisha on lung function and Fractional Exhaled Nitric Oxide (FeNO). The clinical use of FeNO for diagnostic purposes in waterpipe smokers should be taken into account and its correlation with PFTs in clinical context is highly recommended. Furthermore, health officials must implement suitable policies to minimize the use of Shisha smoking.

### 4.5. Study Strengths and Limitations

In this study, we selected a very important topic of current interest, and determined the effects of Shisha smoking on lung function and FeNO. FeNo is novel marker of eosinophilic airway inflammation. While little research exists on Shisha smoking, lung function and FeNO, the literature has failed to consider key physiological parameters including age, height, weight, gender, ethnicity and socio-economical matching. Moreover, we used well-established exclusion criteria to determine the impact of Shisha smoking on lung function and FeNO. The limitations of the present study include lack of specific smoke exposure assessment and the relatively small sample size.

## 5. Conclusions

It is concluded that, lung function parameters FEV1, FEV1/FVC Ratio, FEF-25%, FEF-50%, FEF-75% and FEF-75-85% were significantly decrease in Shisha smokers relative to their control group. There was also a significant reduction in the Fractional Exhaled Nitric Oxide among Shisha smokers compared to control group.
